# Supplementary figures and images for: How riparian and floodplain restoration modify the effects of increasing temperature on adult salmon spawner abundance in the Chehalis River, WA
Source: PLoS One. 2022 Jun 10;17(6):e0268813. doi: 10.1371/journal.pone.0268813 (PMC9187100; doi:10.1371/journal.pone.0268813)

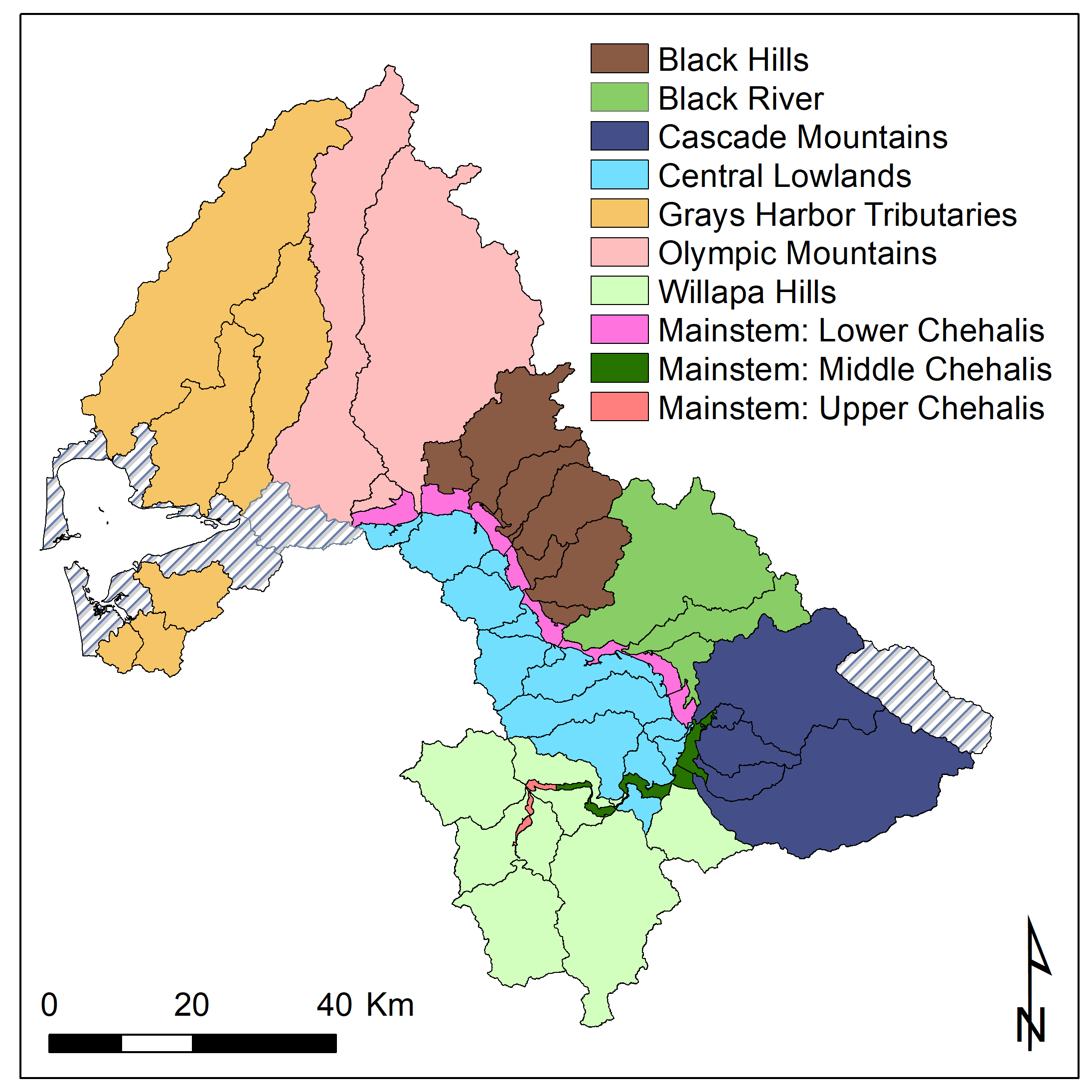

Supplement: S1 Fig — Gray regions are not included in the Ecological Regions. (TIF) [file pone.0268813.s001.tif]

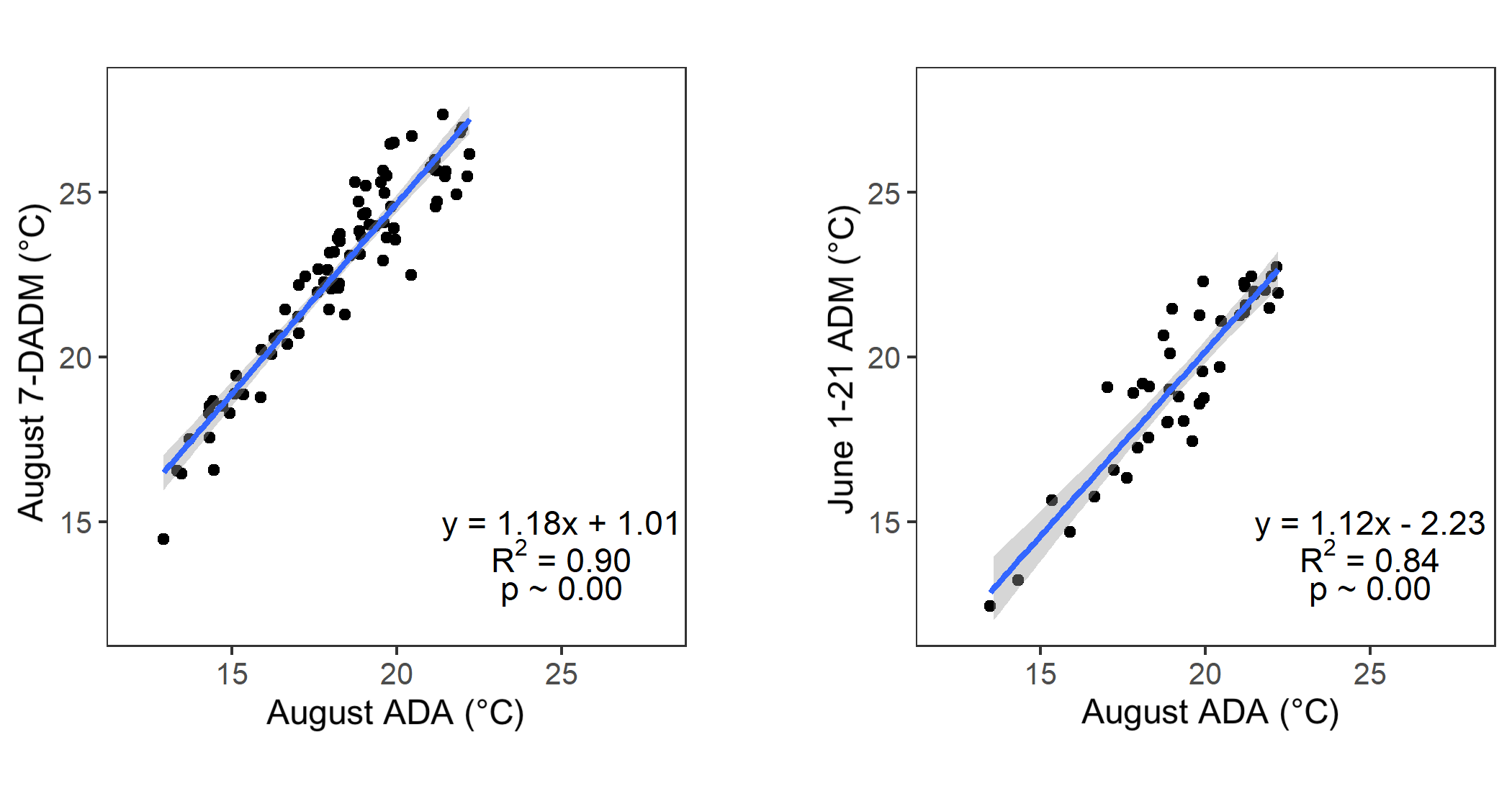

Supplement: S2 Fig — Observed temperature data were used to calculate August ADA (n = 80 sites), 7-DADM (n = 80 sites), and June 1–21 ADM (n = 43 sites). Blue line represents the line of best fit, and grey shading represents the 95% confidence interval. (TIFF) [file pone.0268813.s002.tiff]

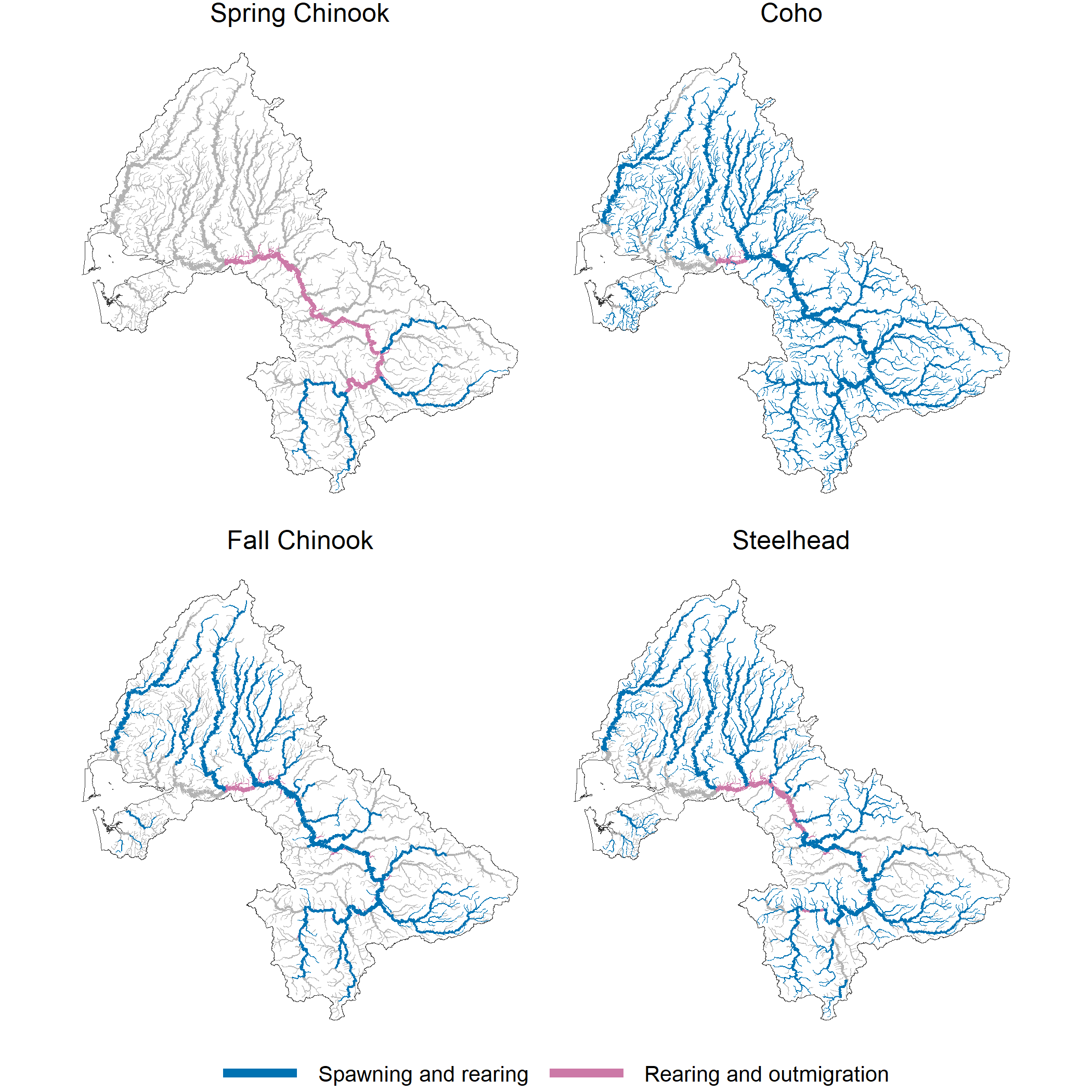

Supplement: S3 Fig — (TIFF) [file pone.0268813.s003.tiff]

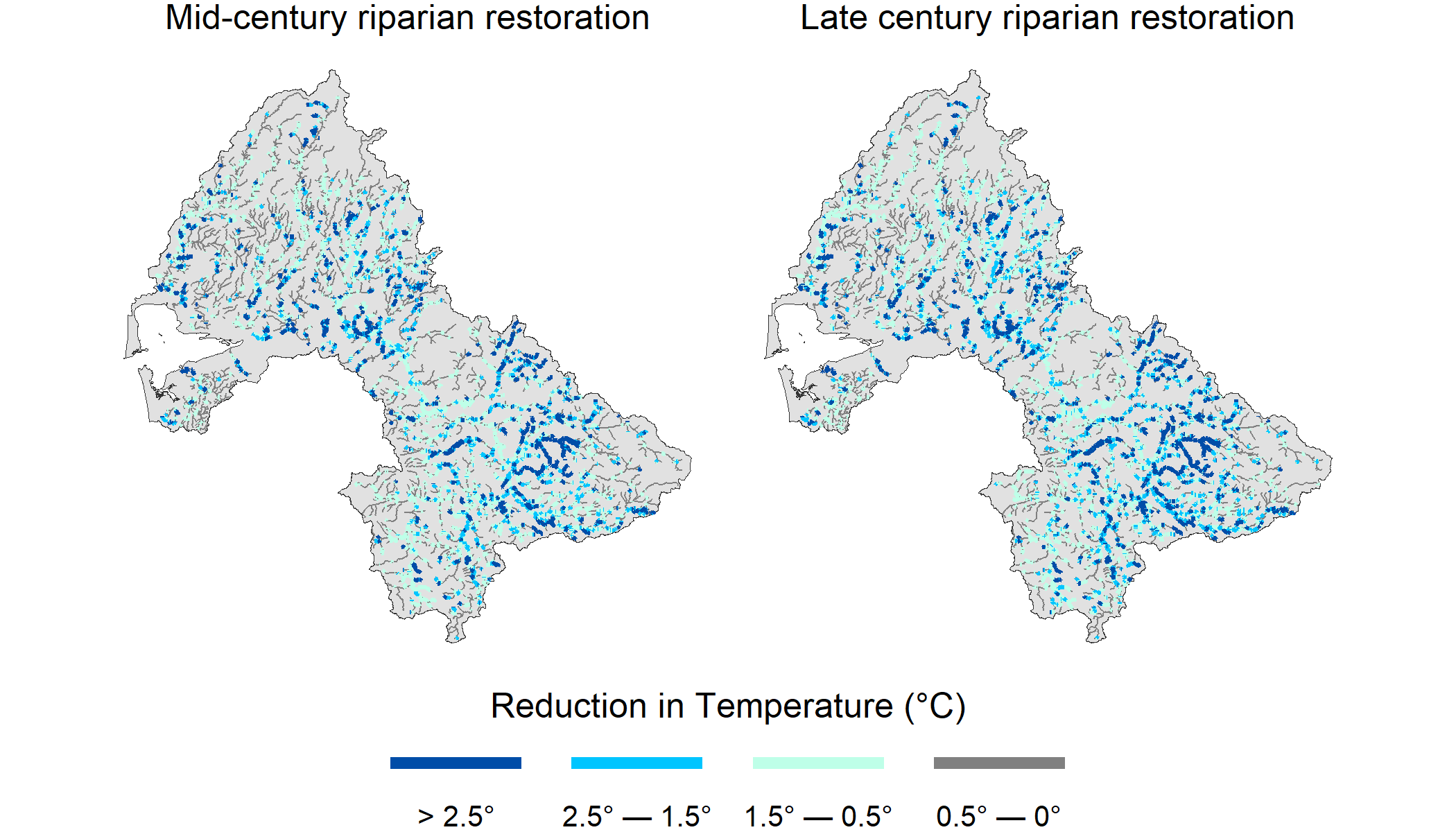

Supplement: S4 Fig — (TIFF) [file pone.0268813.s004.tiff]

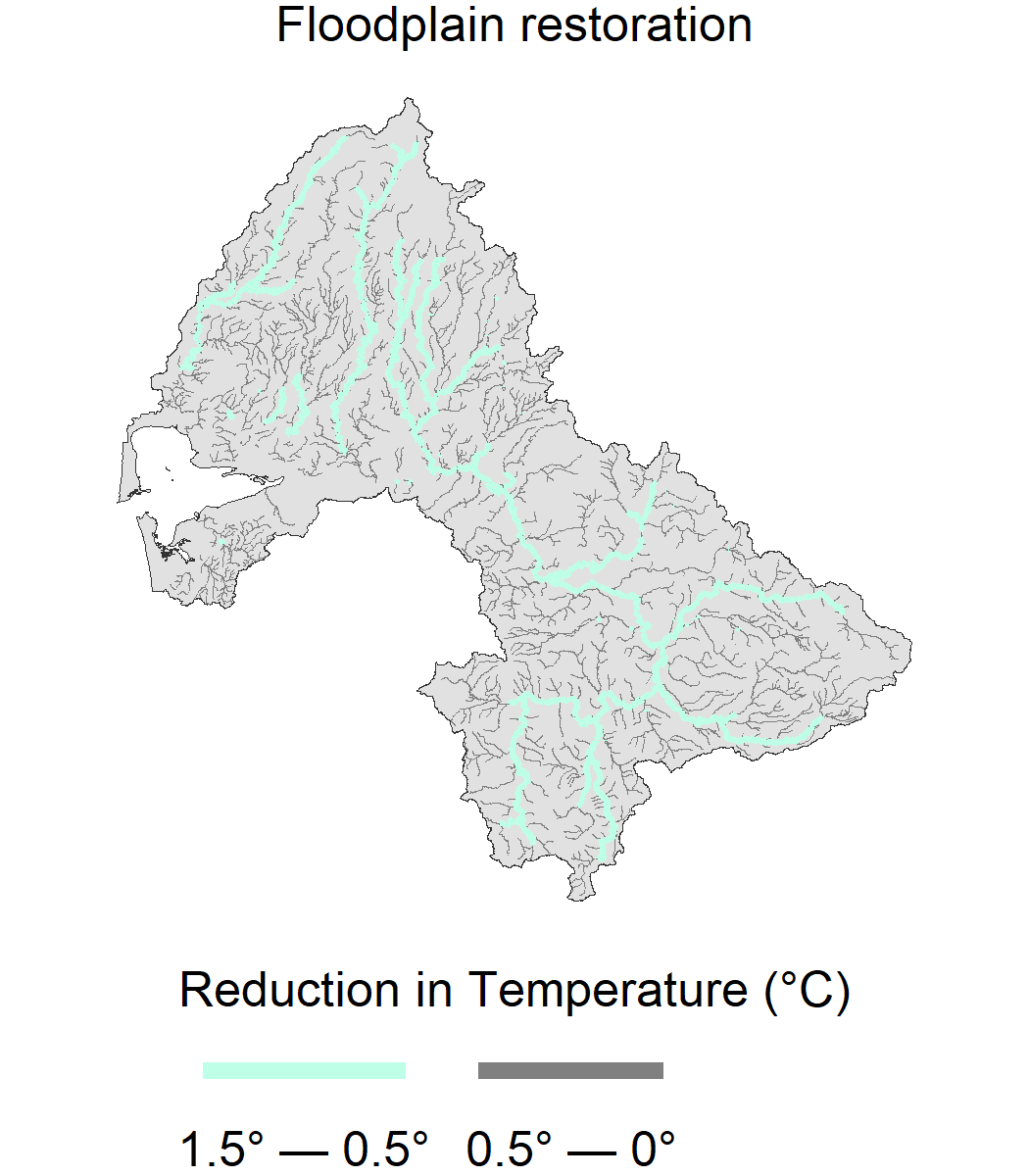

Supplement: S5 Fig — Temperature reduction is the same for both mid-century and late-century. (TIFF) [file pone.0268813.s005.tiff]

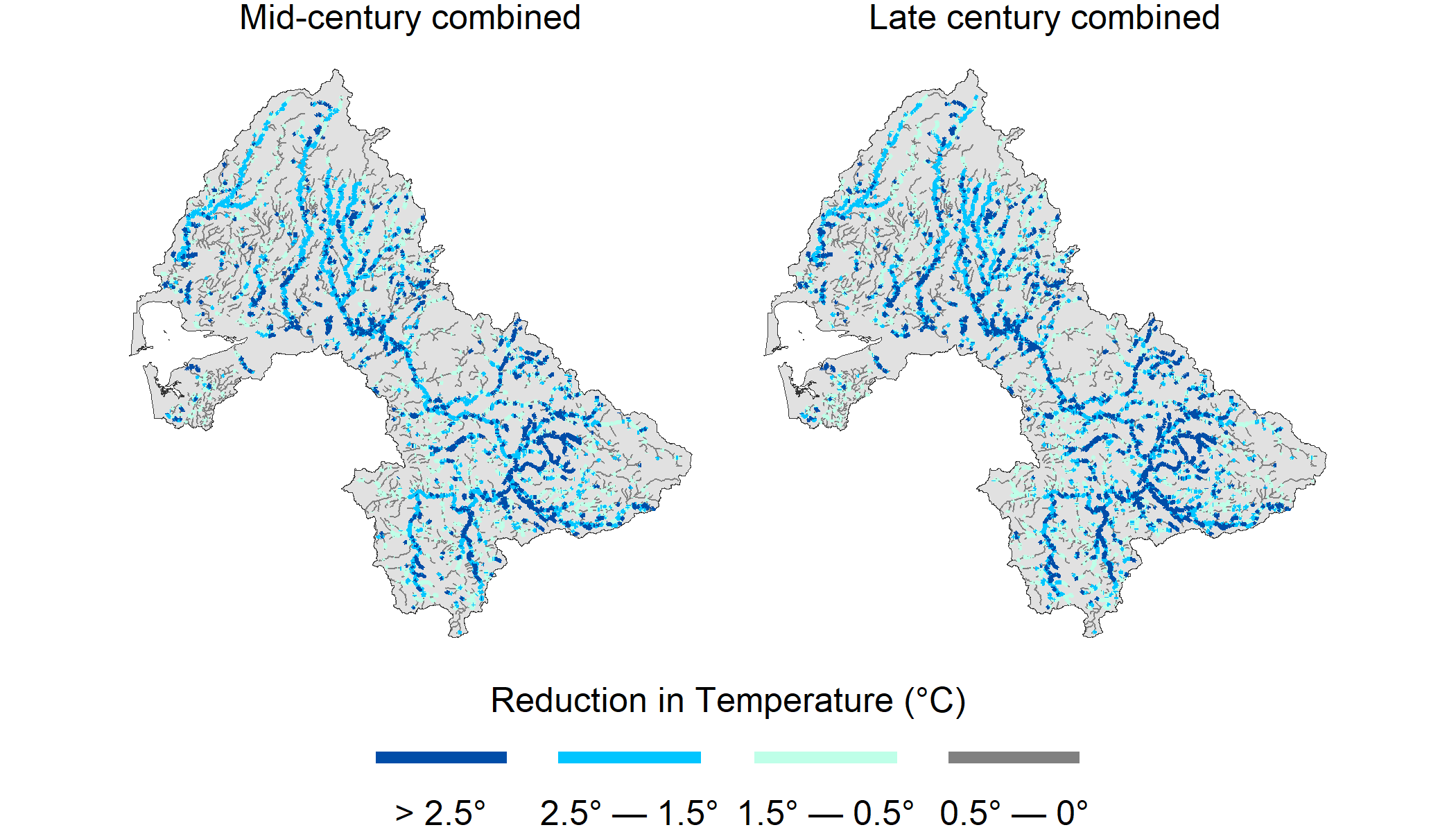

Supplement: S6 Fig — (TIFF) [file pone.0268813.s006.tiff]
